# Unexpected Effects of Oral Isotretinoin in Adolescents With Acne Vulgaris

**DOI:** 10.7759/cureus.17115

**Published:** 2021-08-11

**Authors:** Demet Akpolat

**Affiliations:** 1 Dermatology, Demet Akpolat Clinic, Istanbul, TUR

**Keywords:** isotretinoin, adolescents, acne, hirsutism, hormons

## Abstract

Background

This study aimed to assess the effects of isotretinoin treatment on hirsutism, menstrual cycle and hormonal response in adolescents with acne vulgaris (AV).

Methods

In the study, 76 participants with nodulocystic acne were included. Free testosterone (fT), total testosterone (tT), dehydroepiandrosterone sulfate (DHEAS), luteinizing hormone (LH), follicle-stimulating hormone (FSH), 17-OH progesterone (17-OH PG), and sex hormone-binding globulin (SHBG) levels of the participants were measured before and at the third and sixth months of treatment. Furthermore, the patients were evaluated for hirsutism and menstrual irregularity.

Results

The rates of menstrual irregularity and hirsutism at the beginning and at the third and sixth months of treatment were found to be different (p < 0.05). fT, tT and DHEAS levels at the third and sixth months of treatment were higher than those at the beginning of treatment, and the SHBG level at the sixth month was found to be lower than that at the beginning and third month of treatment (p < 0.05). The tT levels were found to be lower and DHEAS levels were higher than those at the beginning of treatment in patients who presented with menstrual irregularity at the third month of treatment (p < 0.05). The LH and 17-OH PG levels were noted to be lower and DHEAS levels were higher than those at the beginning of treatment in patients who developed hirsutism at the third month of treatment (p < 0.05). The SHBG levels were observed to be lower and DHEAS levels were higher than those before treatment in patients who developed menstrual irregularity at the sixth month of treatment (p < 0.05). SHBG levels were discerned to be lower and DHEAS levels were higher compared to those at the beginning of treatment in patients who developed hirsutism at the sixth month of the treatment (p < 0.05).

Conclusions

Isotretinoin can cause alterations in the adrenal hormone levels. Hirsutism and menstrual irregularity can be observed during treatment follow-ups.

## Introduction

Acne vulgaris (AV) is a chronic inflammatory disease of the pilosebaceous unit (hair follicles and the associated sebaceous glands) that usually occurs during adolescence [1]. The characteristic lesions include open-closed comedones, inflammatory papules, pustules, nodules, and cysts that can cause the formation of scars. Although the etiopathogenesis of AV are not precisely known, it is considered to be multifactorial and includes four main factors: abnormal follicular keratinization, increased sebum production, inflammation, and colonization and activation of Propionibacterium acnes [2,3]. Androgens play an important role in the development and change of sebaceous glands and hair follicles [3,4]. Abnormal expressions of one or several of these factors result in the formation of acne [3,5].

 Isotretinoin (13-cis-retinoic acid) is a synthetic vitamin A derivative used in patients with nodulocystic and resistant acne that does not respond to conventional treatments [6,7]. Most of the side effects of isotretinoin are self-limiting, treatable, and dose-dependent although patients should be monitored regularly by clinicians due to the possibility of serious side effects. There are limited studies on the metabolic and hormonal effects of isotretinoin, which is a relatively new molecule, on humans, and some of them have mentioned conflicting and new side effects [8]. Hirsutism and menstrual irregularity have been detected during the follow-up of patients who received isotretinoin treatment.

Considering the findings of menstrual irregularity and increased hair growth during oral isotretinoin treatment in adults in the literature, we believe that the drug may cause sex hormone changes, hirsutism, and menstrual cycle irregularities in adolescents with AV.

This study aimed to investigate the effects of oral isotretinoin treatment on sex hormones, hirsutism, and menstrual cycle in adolescents with AV.

## Materials and methods

This prospective study was performed with the approval of the Institutional Review Board (Beykoz State Hospital) and in line with the ethical principles of the Declaration of Helsinki. All participants were informed about the study, and a written consent form was obtained from their parents.

In the study, 76 female patients who presented to the dermatology outpatient clinic of Beykoz State Hospital between June 2015 and February 2016 and who met the inclusion criteria were included. The criteria for inclusion in the study were being 14-18 years old and having been clinically diagnosed with nodulocystic acne with a severity of ≥3 according to the Burke and Cunliffe classification [9].

Patients who had menstrual irregularity and increased hair growth before treatment, were pregnant, were using drugs (oral contraceptive pills, injectable hormonal contraception, hormone-containing intrauterine devices or spironolactone) that affect ovarian hormone levels or cause hirsutism, had endocrinopathy, had received oral isotretinoin treatment up to 6 months prior to the study, had liver dysfunction or dyslipidemia, had a contraindication for isotretinoin use, or were not willing to use the medication, drinking alcohol, known disease were excluded from the study.

A form that included anamnesis, dermatological examination, and laboratory tests was developed. Age, age at acne onset, acne severity, age of menarche, and smoking and alcohol use were recorded. The modified Ferriman-Gallwey (mFG) scoring system was used to assess hirsutism [10,11]. Patients diagnosed with nodulocystic acne with a severity of ≥3 according to the Burke and Cunliffe classification were treated with 0.5 mg/kg/day oral isotretinoin. At the beginning of the treatment and at the 3rd and 6th month of treatment, fasting levels of luteinizing hormone (LH), follicle-stimulating hormone (FSH), dehydroepiandrosterone sulfate (DHEAS), 17 hydroxy progesterone (17-OH PG), free testosterone (fT), total testosterone (tT), and sex hormone-binding globulin (SHBG) were measured on the 3rd day of the menstrual cycle. The data obtained were statistically evaluated.

Statistical analysis

 Descriptive statistics were presented using frequency, percentage, mean, and standard deviation values in the analysis of the data. Before treatment and at the third and sixth month of treatment, Cochran's Mantel-Haenszel test statistic was applied to compare the rates of menstrual irregularity and hirsutism. Hormone measurements before treatment and at the third and sixth months of treatment were examined using the repeated analysis of variance. Sidak pairwise comparison test was applied to determine the differences among the timepoints. Mann-Whitney U test was used to examine the hormone levels based on the development of menstrual irregularity and hirsutism at the third and sixth month. All figures' statistical analyses were presented using mean and standard deviation values. The analyses were performed with SPSS 22.0 (SPSS Inc.) statistical program. p values < 0.05 were considered statistically significant.

## Results

The mean age of the patients included in the study was 16.47 ± 1.17 years. The mean age at acne onset was 15.10 ± 1.61 and age of menarche was 11.89 ± 1.37. None of the patients included in the study had any known disease or used systemic medications or alcohol. Of the patients, 5.2% were actively smoking.

 While there was no menstrual irregularity in any of the patients at the beginning of treatment, the issue developed at the third month of treatment in 26 patients (32.2%) and at the sixth month of treatment in 32 patients (42.1%). A statistically significant difference was found among the menstrual irregularity rates of the patients before treatment, at the third month of treatment, and at the sixth month of treatment (p < 0.01). When the mFG scores of the patients were examined, there was no case with a score of >8 at the beginning of treatment, while there were 8 (10.5%) patients with a score of >8 at the third month, and 16 (21.1%) patients with a score of >8 at the sixth month. In the patients, a statistically significant difference was found among the rates of hirsutism before treatment, at the third month of treatment, and at the sixth month of treatment (p < 0.01). 

The mean values for the laboratory parameters of the patients participating in the study before treatment, at the third month of treatment, and at the sixth month of treatment are illustrated in Table 1.

**Table 1:** Hormone levels of the patients according to the months of treatment. LH: luteinizing hormone; FSH: follicle-stimulating hormone; fT: free testosterone; tT: total testosterone; DHEAS: dehydroepiandrosterone sulfate; 17-OH PG: 17-OH progesterone, SHBG: sex hormone-binding globulin. *p < 0.05.

	Before treatment (a)	Third month of treatment (b)	Sixth month of treatment (c)	p	Difference
LH	6.83±7.89	8.01±5.58	6.9±9.85	0.51	-
FSH	3.66±1.83	4.08±1.54	3.74±2.36	0.19	-
fT	1.64±0.5	2.24±0.98	2.23±0.88	0.01*	a-b (p=0.01)* a-c (p=0.01)* b-c (p=0.86)
tT	0.39±0.11	0.63±1.22	0.54±0.2	0,01*	a-b (p=0.01)* a-c (p=0.01)* b-c (p=0.08)
DHEAS	332.8±110.54	360.21±132.2	366.89±117.57	0.01*	a-b (p=0.01)* a-c (p=0.01)* b-c (p=0.06)
17-OH PG	2.31±1.43	2.63±2.97	2.52±1.71	0.44	-
SHBG	51.45±20.62	56.7±38.55	41.31±17.43	0.01*	a-b (p=0.16) a-c (p=0.01)* b-c (p=0.01)*

**Figure 1:**
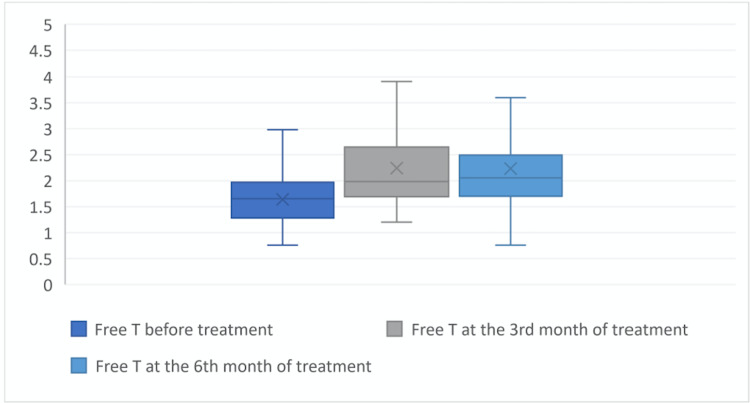
Free testosterone level before the treatment, at the third month of treatment, and at the sixth month of treatment. X symbol: defines mean value.

**Figure 2:**
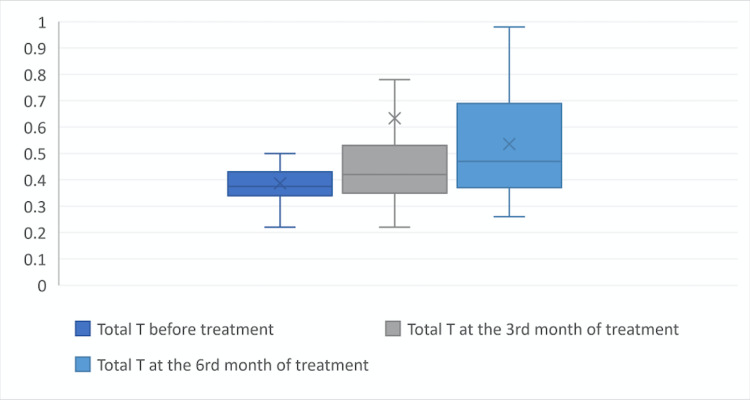
Total testosterone level before the treatment, at the third month of treatment, and at the sixth month of treatment. X symbol: defines mean value.

**Figure 3:**
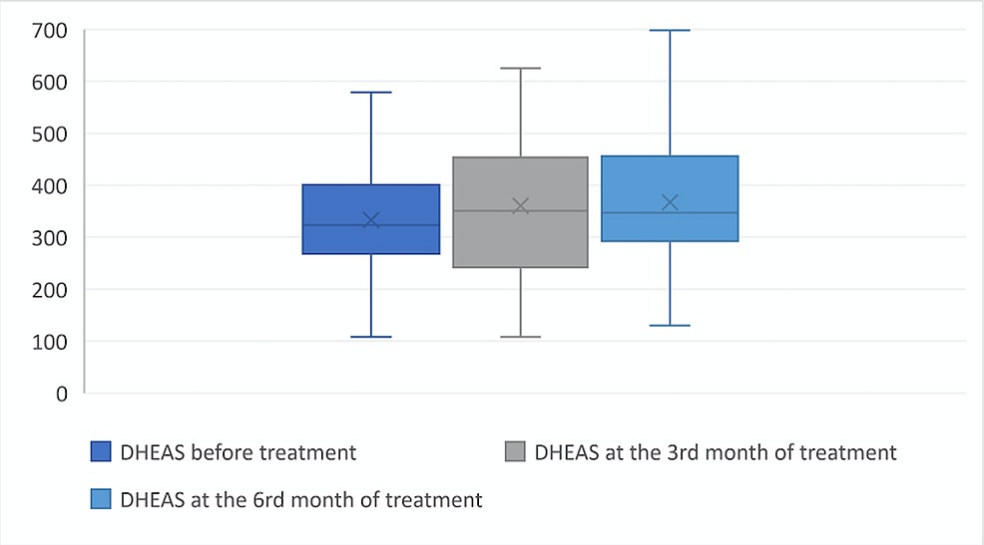
Dehydroepiandrosterone sulfate level before the treatment, at the third month of treatment, and at the sixth month of treatment. DHEAS: dehydroepiandrosterone sulfate X symbol: defines mean value.

At the beginning of the treatment the values of fT, tT and DHEAS were 1.64±0.5, 0.39±0.11 and 3328±110.54, at the third month of treatment were 2.24±0.98, 0.63±1.22 and 360.21±132.2 ant at the sixth month of treatment were 2.23±0.88, 0.54±0.2 and 366.89±117.57, respectively. When the laboratory parameters at the beginning, third month of treatment, and sixth month of treatment were compared, it was found that fT, tT, and DHEAS levels at the third and sixth month of treatment were statistically significantly higher than at the beginning of treatment (p = 0.01) (Figures 1, 2, 3).

**Figure 4:**
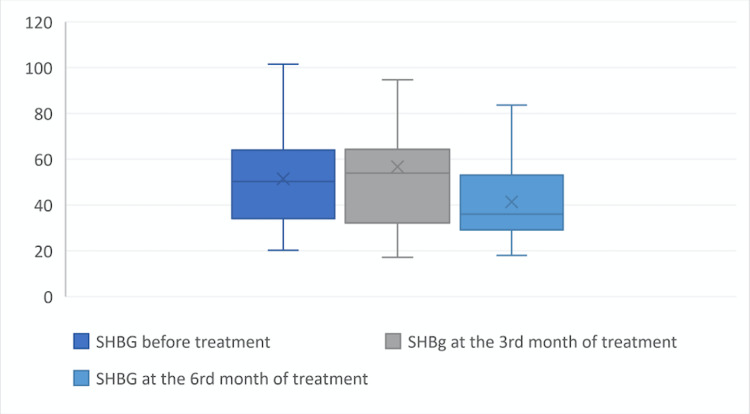
SHBG level before the treatment, at the third month of treatment, and at the sixth month of treatment. SHBG: sex hormone-binding globulin. X symbol: defines mean value.

However, the SHBG level at the 6th month of treatment was statistically significantly lower than at the beginning of treatment and at the third month of treatment (p = 0.01), (Figure 4; Table 2). In terms of FSH (p = 0.19), LH (p = 0.51), and 17-OH PG (p = 0.44) levels, there was no statistically significant difference between the third and sixth month and the beginning of the treatment (Table 2).

In women who developed menstrual irregularity at the third month of treatment, tT levels were found to be statistically significantly lower and DHEAS levels were found to be statistically significantly higher compared to the beginning of the treatment (p < 0.05). In women who developed menstrual irregularity at the third month of treatment, there was no statistically significant difference among the LH, FSH, fT, 17-OH PG, and SHBG levels compared to the beginning of the treatment (p > 0.05). In women who developed hirsutism at the third month of treatment, LH and 17-OH PG levels were found to be statistically significantly lower and DHEAS levels were found to be statistically significantly higher than at the beginning of the treatment (p < 0.05). In women who developed hirsutism at the third month of treatment, there was no statistically significant difference among the FSH, fT, tT, and SHBG levels compared to the beginning of the treatment (p > 0.05) (Table 2).

**Table 2:** The relationship between hormone levels and menstrual irregularity and hirsutism at the third month of treatment. LH: luteinizing hormone; FSH: follicle-stimulating hormone; fT: free testosterone; tT: total testosterone; DHEAS: dehydroepiandrosterone sulfate; 17-OH PG: 17-OH progesterone; SHBG: sex hormone-binding globulin. *p < 0.05.

		LH	FSH	fT	tT	DHEAS	17-OH PG	SHBG
Menstrual irregularity	No	8.05±5.69	3.99±1.46	2.33±1.08	0.72±1.49	310.99±94.3	2.75±3.37	65.54±43.15
	Yes	7.94±5.49	4.25±1.7	2.09±0.76	0.46±0.14	454.86±144.36	2.38±2.03	39.69±18.8
	p	0.18	0.23	0.11	0.03*	0.01*	0.35	0.19
Hirsutism	No	8.26±5.72	4.03±1.56	2.21±1.01	0.64±1.29	348.48±129.26	2.71±3.12	57.38±40.2
	Yes	5.94±3.85	4.45±1.45	2.55±0.76	0.6±0.15	459.88±121.06	1.95±0.86	50.9±20.34
	p	0.04*	0.35	0.24	0.32	0.01*	0.04*	0.26

In women who developed menstrual irregularity at the sixth month of treatment, SHBG levels were found to be statistically significantly lower and DHEAS levels were found to be statistically significantly higher than at the beginning of the treatment (p < 0.05). On the other hand, in women who developed menstrual irregularity at the sixth month of treatment, there was no statistically significant difference in the LH, FSH, fT, 17-OH PG, and tT levels compared to the beginning of the treatment (p > 0.05). In women who developed hirsutism at the sixth month of treatment, there was no statistically significant difference in the LH, FSH, fT, tT, and 17-OH PG levels compared to the beginning of the treatment (p > 0.05). On the other hand, in women who developed hirsutism at the sixth month of treatment, SHBG levels were found to be statistically significantly lower and DHEAS levels were found to be statistically significantly higher than at the beginning of the treatment (p < 0.05) (Table 3).

**Table 3:** The relationship between hormone levels and menstrual irregularity and hirsutism at the sixth month of treatment, LH: luteinizing hormone; FSH: follicle-stimulating hormone; fT: free testosterone; tT: total testosterone; DHEAS: dehydroepiandrosterone sulfate; 17-OH PG: 17-OH progesterone; SHBG: sex hormone-binding globulin. *p < 0.05.

		LH	FSH	fT	tT	DHEAS	17-OH PG	SHBG
Menstrual irregularity	No	7.63±12.7	3.98±2.57	1.91±0.57	0.49±0.20	316.73±76.6	2.85±2.05	46.37±18.24
	Yes	5.88±3.27	3.42±1.11	2.68±1.04	0.59±0.20	435.86±129.7	2.06±0.95	34.34±13.68
	p	0.15	0.30	0.10	0.13	0.02*	0.09	0.03*
Hirsutism	No	6.83±10.5	3.78±2.80	2.08±0.80	0.51±0.21	354.24±112.2	2.50±1.91	43.73±18.59
	Yes	7.13±7.11	3.59±2.99	2.79±0.99	0.63±0.13	414.31±128.7	2.58±0.56	32.21±6.95
	p	0.22	0.48	0.13	0.08	0.04*	0.43	0.04*

## Discussion

In our study, we found that oral isotretinoin caused hirsutism and menstrual irregularity and changes in fT, tT, and SHBG levels at the third and sixth month of treatment.

AV is a disease of the pilosebaceous unit and mostly occurs during adolescence. Increased production of androgen-mediated sebum with puberty and follicular hyperkeratinization play an important role in the etiopathogenesis of AV [12]. Androgens are mainly synthesized in the ovaries and adrenal gland in women. DHEAS, androstenedione, and testosterone are serum androgens. LH and FSH are gonadotropins secreted by the pituitary gland, and they regulate steroid synthesis.

Studies have examined the effects of oral isotretinoin treatment on pituitary and adrenal hormones and have yielded different results. In a study by Karadağ et al. investigating the effects of isotretinoin treatment on the pituitary-adrenal axis, it was found that three months of oral isotretinoin treatment reduced LH, fT, tT, and DHEAS levels [13]. A study by Koçyiğit et al. found an increase in tT levels and a decrease in DHEAS levels three months after oral isotretinoin treatment in acne patients when compared to the control group [14]. In a study by Çetinözman et al., it was reported that oral isotretinoin improved acne lesions without causing any androgen imbalance [15]. In a study by Açmaz et al., although there was a significant decrease in fT levels, there was no significant difference in FSH, LH, DHEAS, SHBG, and tT levels 6 months after isotretinoin treatment [16]. In our study, in the tests performed in the follicular phase of the menstrual cycle at the 3rd and 6th month of treatment, an increase in fT, tT, and DHEAS levels and a decrease in SHBG level were detected when compared to pre-treatment levels; however, there was no significant difference in FSH, LH, and 17-OH PG levels.

Hirsutism refers to an increase in terminal hair in women. Although it is often one of the clinical signs of hyperandrogenemia, androgen levels are within normal limits in some affected women. An mFG score of >8 is considered to signify hirsutism. In a study by Demirci et al., hypertrichosis was detected in 11 of 56 patients at the 6th month of oral isotretinoin treatment. According to the Ferriman-Gallwey (FG) score, only one patient was accepted as a case of hirsutism [17]. In a study by Aktar et al., FG score at the third month of treatment was found to be significantly higher than that before treatment. Clinically detected hypertrichosis was observed in 11 patients [18]. In our study, eight patients at the third month of treatment and 16 patients at the sixth month of treatment were found to have an mFG score of >8 compared to the beginning of treatment, and they were accepted as having hirsutism. It was believed that this clinical finding might be because oral isotretinoin causes decreased levels of SHBG and increased levels of circulating fT, tT, and DHEAS as well as increased androgen receptor sensitivity.

The menstrual cycle depends on the balance and normal blood levels of many hormones. It has been suggested that retinoids play an important role in endometrial growth and secretory differentiation, and lower serum vitamin A levels were found in patients suffering from menorrhagia compared to healthy people [19]. Retinoids have been shown to inhibit endometrial fibroblast and epithelial cell proliferation [20]. In a study by Aktar et al., menstrual irregularity was detected in 33.3% of the cases at the third month of treatment [18]. In the study by Karadağ et al., menstrual irregularity was observed in 28.8% of the cases during oral isotretinoin treatment [13]. In a study by Kwon et al., it was reported that 20% of the 40 patients who received oral isotretinoin treatment for at least four months at a dose range of 0.7-1.2 mg/kg/day developed menstrual irregularity; however, the menstrual cycle of all cases returned to normal after the treatment was discontinued [21]. In our study, similar to the literature, 26 patients developed menstrual irregularity at the third month of treatment and 32 patients at the sixth month of treatment. It was observed that the DHEAS levels of the patients who developed menstrual irregularity at the third and sixth month of treatment were higher than those of the patients who did not develop menstrual irregularity. Besides, the SHBG levels of the patients who developed irregularity at the 6th month were lower than those of the patients who did not develop menstrual irregularity.

Our study has certain limitations. First of all, patients with a clinical acne severity <3 according to the Burke and Cunliffe classification could not be evaluated. The reason for this omission was the insufficient number of patients belonging to this patient group and the homogenization problem that could arise if those patients had been included. Use of the oral contraceptive pill was one of the study exclusion criteria. However, in other studies to be planned, the effects of oral isotretinoin on menstrual cycle, hirsutism and hormone panel can be compared in patient groups using and not using oral contraceptives. Another shortcoming of our study is that we did not evaluate the effects of oral isotretinoin on the sex hormone panel of male patients with severe acne, since we included female patients in our study. Also the cumulative dose used in the treatment was taken as a single value of 0.5 mg/kg/day. Treatment could be followed up with simultaneous low doses, and it could be evaluated whether there was a change in the dose-dependent findings. However, high doses were planned to be used to ensure treatment success since our patient group was a severe nodulocystic acne group.

## Conclusions

Patients with no complaints of menstrual irregularity or hirsutism before treatment showed signs of hyperandrogenism during treatment follow-up. Hence, although isotretinoin decreases sebum expression owing to its anti-androgenic effects, it may cause a decrease in SHBG levels and an increase in the amount of free androgens, thus leading to hirsutism and menstrual irregularity. Further investigations are required to prove the mechanisms by which oral isotretinoin exerts its variable effects on the menstrual cycle, hirsutism, and hormones.
